# Supplementary material for: U2-Net and ResNet50-Based Automatic Pipeline for Bacterial Colony Counting
Source: Microorganisms. 2024 Jan 18;12(1):201. doi: 10.3390/microorganisms12010201 (PMC10820204; doi:10.3390/microorganisms12010201)
Supplement: Supplementary file 1 [file microorganisms-12-00201-s001.zip › microorganisms-2781479-supplementary.pdf]

microorganisms

Supplementary Materials

Table S1. CMOS parameters.

Items	Parameters
Brand and model	SHL- U3CMOS05100KPA/TP105100A; https://shunhuali.com/product/229.html
Image sensor circuit	1/2.5 inch; CMOS; Color
Effective pixels	5.1 Mega pixels
Pixel sizes	2.2 × 2.2 μm
Frame-rates/Resolution	14.2 fps/2560 × 1922 ppi
Lens mount	C-Mount
Signal-to-noise ratio	38.5 dB
Dynamic range	67.74 dB
Sensitivity	1.76 v/lux-sec
Spectral range	380–650 nm (with an IR-cut filter)
Support systems	Windows; Linux; macOS; or Android
Data interface	USB

Table S2. Len parameters.

Items	Parameters
Brand and model	SHL; https://shunhuali.com/product/188.html
Focal length	2.8-12 mm
Chart size	1/2 inch
Pixel	3 Mega pixels
Aperture value	F1.6 (IR)
Mount	C-Mount
Back focal length	17.53 mm
Flange back length	9 mm
Minimum object distance	0.3 m

Table S3. Comparison of colony counting between ResNet50, Watershed, and a reference CNN algorithm [1].

Class	A proposed CNN		Watershed		ResNet50	
	Precision	Recall	Precision	Recall	Precision	Recall
2	0.93	0.92	0.67	0.57	0.90	0.97
3	0.83	0.88	0.51	0.48	0.95	0.84
4	0.77	0.70	0.37	0.37	0.91	0.89
5	0.59	0.44	0.24	0.26	0.86	0.90
6	0.71	0.73	0.21	0.45	0.96	0.88

Table S4. Recovery of our approach on the colony counting of eight different bacterial species.

Colony type	*E. coli*	*L. ivanovii*	*L. monocytogenes*	*S. aureus*	*S. epidermidis*	*S. typhimurium*	*Shigella*	*V. parahaemolyticus*
Recovery	97.00%	99.77%	99.99%	94.07%	98.39%	99.95%	99.30%	99.13%

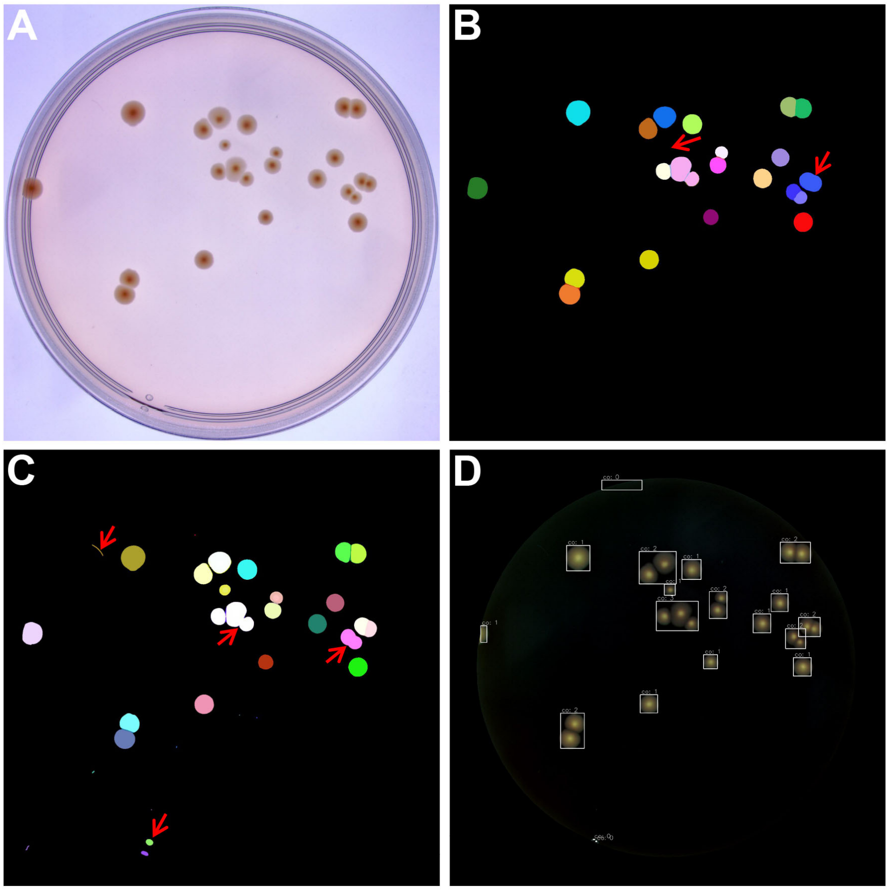

Figure S1. Comparison of the proposed method and SAM. (**A**) A raw colony image; (**B**) The result taking 31 seconds based on default parameters for SAM; (**C**) The result taking 411 seconds with carefully selected parameters for SAM; (**D**) The result taking 38 seconds based on default parameters for our approach. The red arrows represent identification of non-target objects, missed colony recognition, and inability to classify adhesive colonies. The default parameters for SAM are as follows: points_per_side: 32; points_per_batch: 64; pred_iou_thresh: 0.88; stability_score_thresh: 0.95; stability_score_offset: 1.0; box_nms_thresh: 0.7; crop_n_layers: 0; crop_nms_thresh: 0.7; crop_overlap_ratio: 0.3413; crop_n_points downscale_factor: 1; point_grids: None; min_mask_region_area: 0; output_mode: "binary_mask". To enhance the accuracy of SAM in recognizing colonies, we modified the following parameters: points_per_side: 128; crop_n_layers: 3.

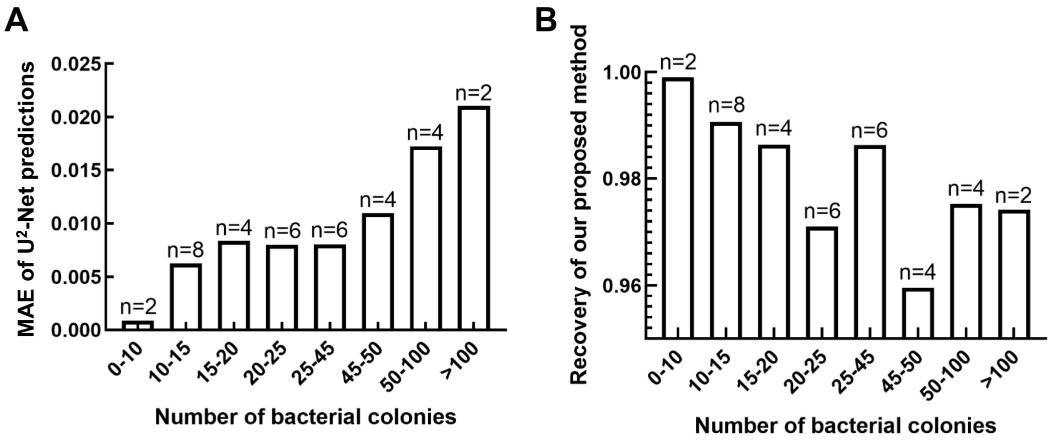

Figure S2. Increase in colony aggregation or the size of the colonies has a minor impact on colony counting. (**A**) Comparison of the MAE performance of U^2-Net on images with different colony quantity ranges. (**B**) Comparison of the recovery performance of the entire counting process on images with different colony quantity ranges. The sample sizes for each colony quantity range are annotated on the bars in the histogram.

References

1. Ferrari, A.; Lombardi, S.; Signoroni, A. Bacterial colony counting with Convolutional Neural Networks in Digital Microbiology Imaging. *Pattern Recognit* **2017**, *61*, 629–640. https://doi.org/10.1016/j.patcog.2016.07.016.
